# Neuroenergetic response to prolonged cerebral glucose depletion after severe brain injury and the role of lactate

**DOI:** 10.1186/cc14528

**Published:** 2015-03-16

**Authors:** C Patet, H Quintard, T Suys, L Pellerin, P Magistretti, M Oddo

**Affiliations:** 1 CHUV - Lausanne University Hospital, Lausanne, Switzerland; 2 University of Lausanne, Switzerland; 3 Brain Mind Institute, Lausanne, Switzerland

## Introduction

In patients with acute brain injury (ABI), increased cerebral energy demand is frequent, potentially leading to cerebral glucose depletion (GD) and poor outcome. In this setting, lactate may act as supplemental fuel. We examined dynamics of cerebral lactate supply during prolonged GD in ABI.

## Methods

We retrospectively analyzed severe ABI (18 TBI, eight SAH) monitored with brain oxygen and cerebral microdialysis (CMD) to measure hourly levels of cerebral extracellular glucose, lactate, pyruvate and lactate/pyruvate ratio (LPR). Variations of CMD variables were analyzed as a function of GD (defined as spontaneous decreases of CMD glucose from normal to low (<1.0 mM), at least 2 hours) and increased cerebral energy demand (LPR >25).

## Results

**Figure 1:**
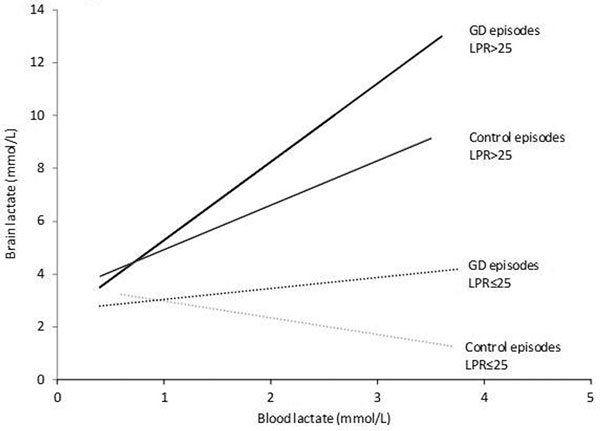
Correlations between blood and brain lactate

During GD (60 episodes; 26 patients), we found an increase of CMD lactate (4 ± 2.3 vs. 5.4 ± 2.9 mM) and LPR (27 ± 6 vs. 35 ± 9; all *P *< 0.005) while brain oxygen and blood lactate remained normal. Dynamics of lactate and glucose supply were studied by analyzing the relationship between blood and CMD samples. No correlation between blood and brain lactate was found when brain glucose and LPR were normal (*r *= -0.12, *P *= 0.48; Figure 1), while this correlation became linear during GD, progressively rising to *r *= 0.53 (*P *< 0.0001) when energy demand increased, suggesting increased cerebral lactate availability. The correlation between blood and brain glucose changed in the opposite direction, decreasing from *r *= 0.78 to 0.37 (*P *< 0.0001) during GD and at LPR >25.

## Conclusion

Energy dysfunction is associated with increased supply of nonhypoxic cerebral lactate. Our data suggest lactate may act as alternative substrate after ABI when availability of cerebral glucose is reduced.

